# Supplementary material for: Impact of precisely-timed inhibition of gustatory cortex on taste behavior depends on single-trial ensemble dynamics
Source: eLife. 2019 Jun 24;8:e45968. doi: 10.7554/eLife.45968 (PMC6625792; doi:10.7554/eLife.45968)
Supplement: Transparent reporting form [file elife-45968-transrepform.pdf]

eLIFE
1st Floor
24 Hills Road
Cambridge CB2 1JP, UK
P 01223 855340
W elifesciences.org
T @elife

eLife's transparent reporting form

We encourage authors to provide detailed information *within their submission* to facilitate the interpretation and replication of experiments. Authors can upload supporting documentation to indicate the use of appropriate reporting guidelines for health-related research (see EQUATOR Network), life science research (see the BioSharing Information Resource), or the ARRIVE guidelines for reporting work involving animal research. Where applicable, authors should refer to any relevant reporting standards documents in this form.

If you have any questions, please consult our Journal Policies and/or contact us: editorial@elifesciences.org.

Sample-size estimation

- You should state whether an appropriate sample size was computed when the study was being designed
- You should state the statistical method of sample size computation and any required assumptions
- If no explicit power analysis was used, you should describe how you decided what sample (replicate) size (number) to use

Please outline where this information can be found within the submission (e.g., sections or figure legends), or explain why this information doesn't apply to your submission:

Our study follows up on more than 10 publications (from several research groups) about single neuron coding of tastes in primary sensory cortex. As such, we first replicate the (already published) basic features of the single neuron cortical taste code (Figure 3) with a sample of neurons (>300 single units) that is at least twice as large as any of the previous studies. In the rest of our study, we investigate novel effects that have never been observed and in addition, do so through "within-session" analyses (that is, only a subset of trials in a session was used in each analysis). While within-session analyses have the potential of winnowing down the effective number of trials being analyzed, they also ensure that we (purposefully) focus only on the most substantial effects produced by our manipulation.

Replicates

- You should report how often each experiment was performed
- You should include a definition of biological versus technical replication
- The data obtained should be provided and sufficient information should be provided to indicate the number of independent biological and/or technical replicates
- If you encountered any outliers, you should describe how these were handled
- Criteria for exclusion/inclusion of data should be clearly stated
- High-throughput sequence data should be uploaded before submission, with a private link for reviewers provided (these are available from both GEO and ArrayExpress)

Please outline where this information can be found within the submission (e.g., sections or figure legends), or explain why this information doesn't apply to your submission:

Details about replication/technical robustness are in the Materials and Methods section. We applied Bayesian statistical techniques in our analyses (specific models are detailed in the Materials and Methods section) that are robust to outliers and avoided excluding data from our analyses.

Statistical reporting

- Statistical analysis methods should be described and justified
- Raw data should be presented in figures whenever informative to do so (typically when N per group is less than 10)
- For each experiment, you should identify the statistical tests used, exact values of N, definitions of center, methods of multiple test correction, and dispersion and precision measures (e.g., mean, median, SD, SEM, confidence intervals; and, for the major substantive results, a measure of effect size (e.g., Pearson's r, Cohen's d)
- Report exact p-values wherever possible alongside the summary statistics and 95% confidence intervals. These should be reported for all key questions and not only when the p-value is less than 0.05.

Please outline where this information can be found within the submission (e.g., sections or figure legends), or explain why this information doesn't apply to your submission:

Details of statistical methods are in the Materials and Methods section. We used approaches from Bayesian statistics throughout the study – the statistical models used have been elucidated in detail in the Materials and Methods section. Summary statistics (in the Results section) describe results in terms of 95% Bayesian credible intervals (and not confidence intervals). Credible intervals inherently act as tests of statistical significance, and have been used as such throughout the study.

(For large datasets, or papers with a very large number of statistical tests, you may upload a single table file with tests, Ns, etc., with reference to sections in the manuscript.)

Group allocation

- Indicate how samples were allocated into experimental groups (in the case of clinical studies, please specify allocation to treatment method); if randomization was used, please also state if restricted randomization was applied
- Indicate if masking was used during group allocation, data collection and/or data analysis

Please outline where this information can be found within the submission (e.g., sections or figure legends), or explain why this information doesn't apply to your submission:

Our study involves using within-subject controls, such that every animal in the study experiences all the conditions of the experiment. Details on the experimental set up and study design are provided in the Materials and Methods section.

Additional data files ("source data")

- We encourage you to upload relevant additional data files, such as numerical data that are represented as a graph in a figure, or as a summary table
- Where provided, these should be in the most useful format, and they can be uploaded as "Source data" files linked to a main figure or table
- Include model definition files including the full list of parameters used
- Include code used for data analysis (e.g., R, MatLab)
- Avoid stating that data files are "available upon request"

Please indicate the figures or tables for which source data files have been provided:

Being dense recordings of large ensembles of neurons, our data files are usually very large in size (running into tens of GBs). We have, therefore, made our data accessible on a public fileshare system maintained by Library and Technology Services (LTS) in Brandeis University. We can give any interested researchers access to the datasets through Brandeis LTS, but the files are prohibitively large to be hosted on a more general file-share platform.
